# Analysis of Genes Involved in Oxidative Stress and Iron Metabolism in Heart Failure: A Step Forward in Risk Stratification

**DOI:** 10.7759/cureus.60707

**Published:** 2024-05-20

**Authors:** Pedro X Silva, Laura Aguiar, Marcos Gaspar, Paula Faustino, Luiz M Falcão, Mário Barbosa, Manuel Bicho, Ângela Inácio

**Affiliations:** 1 Genetics Laboratory, Faculty of Medicine of the University of Lisbon, Lisbon, PRT; 2 Associate Laboratory TERRA, Environmental Health Institute, Faculty of Medicine of the University of Lisbon, Lisbon, PRT; 3 Department of Genetics, Bento da Rocha Cabral Institute for Scientific Research, Lisbon, PRT; 4 Department of Human Genetics, National Institute of Health Dr. Ricardo Jorge, Lisbon, PRT; 5 Cardiovascular Centre of the University of Lisbon (CCUL@RISE), Faculty of Medicine of the University of Lisbon, Lisbon, PRT; 6 Department of Medicine, Hospital Lusíadas Lisboa, Lisbon, PRT

**Keywords:** personalized medicine, epistasis, oxidative stress, iron metabolism, genetics, heart failure

## Abstract

Introduction: Heart failure (HF) is a clinical syndrome characterized by cardinal symptoms that may be accompanied by signs. It results from structural and/or functional abnormalities of the heart leading to elevated intracardiac pressures and/or inadequate cardiac output at rest and/or during exercise. The prevalence of iron deficiency and anemia justifies the current guidelines recommendation of screening. Genes *HP*, *ACE*, *MTHFR, HFE,* and *CYBA* are involved in oxidative mechanisms, iron metabolism, and hematologic homeostasis. This study investigates the contribution of variants Hp1/2 (*HP*), I/D (*ACE*), C677T (*MTHFR*), C282Y and H63D (*HFE*), and C242T (*CYBA*) to the development of HF, either independently or in epistasis.

Methods: We used a database of 389 individuals, 143 HF patients, and 246 healthy controls. Genotypes were characterized through PAGE electrophoresis, PCR, PCR-RFLP, and multiplex-ARMS. Data analysis was performed with the SPSS® 26.0 software (IBM Corp., Armonk, NY).

Results: We observed a significant association between the *MTHFR *gene and HF predisposition. The presence of allele T and genotype CT constituted risk, while genotype CC granted protection. Epistatic interactions revealed risk between genotype II of the *ACE* gene and genotypes CC (*C282Y)* or HH (*H63D) *of the* HFE *gene. Risk was also observed for interactions between genotype CC (*CYBA*)and genotypes 2-2 (*HP)*, CT (*MTHFR*), or HH (*HFE-H63D)*.

Conclusion: We concluded that genes *HP*, *ACE*, *MTHFR, HFE,* and *CYBA* contribute to the susceptibility for HF, individually or in epistasis. This study contributes to the clarification of the role that genes involved in oxidative mechanisms and iron metabolism play in the physiopathology of HF. It is, therefore, a step forward in risk stratification and personalized medicine.

## Introduction

Heart failure (HF) is a complex clinical syndrome arising secondary to abnormalities in cardiac structure and/or function, whether inherited or acquired, resulting in elevated intracardiac pressures and/or inadequate cardiac output at rest and/or during exercise. While genotypes cannot be altered, comprehending the association between genotypes and HF is crucial for assessing the risk of disease development and adopting early preventive strategies, given that the cardiovascular risk continuum commences with the genetic heritage.

In this study, we aimed to broaden the knowledge regarding the role of specific genes involved in oxidative metabolism, and iron and hematologic homeostasis, along with their epistatic interactions, in HF development.

The presence and production of reactive oxygen species (ROS) modify numerous cardiovascular functions, affecting cell proliferation, migration, and death [1]. Consequently, oxidative stress is highly implicated in cardiovascular events such as HF, ischemia, and reperfusion and acute phenomena like myocardial infarction. Simultaneously, diseases like anemia, although rarely solely responsible for HF development in a normal heart, can rapidly decompensate a structural cardiopathy, causing volume overload and worsening cardiac function through compensatory tachycardia and increased cardiac output. Studies have shown that HF patients with anemia exhibit increased resistance to therapy, higher hospitalization rates, and elevated mortality compared to HF patients without anemia [2,3].

Iron, an essential component of hemoglobin, plays a key role in erythropoiesis, serving as a cofactor for oxygen storage and forming proteins involved in cardiac muscle cell metabolism, such as the ones being part of oxidative enzymes from the mitochondrial respiratory chain.

The systemic metabolism of iron is regulated by the hormone hepcidin. The levels of hepcidin are chronically elevated in proinflammatory conditions like HF, disrupting iron homeostasis and causing iron deficiency. Not only can an ongoing HF cause iron deficiency, but iron deficiency also seems to be capable of inducing HF [4]. Iron deficiency is common in stable chronic HF patients with a prevalence of around 30 to 50%, irrespective of anemia or hematologic abnormalities [4]. It is associated with higher mortality, lower functional capacity [5], increased hospitalization, and early readmission [4]. Iron is essential for the effective activity of the ROS-scavenging enzymes. A study showed a reduced expression of the ROS-scavenging enzymes glutathione peroxidase (by 21%), superoxide dismutase (by 20%), and catalase (by 23%) in patients with HF and myocardial iron deficiency, thereby closely correlating the metabolism of iron with oxidative stress [6].

According to the current European Society of Cardiology (ESC) guidelines for HF, the assessment of iron deficiency and anemia is recommended in HF patients. Intravenous iron supplementation should be considered in patients with left ventricular ejection fraction (LVEF) < 45% and iron deficiency to improve symptoms, as well as in patients recently hospitalized for HF and with LVEF < 50% and iron deficiency to reduce the risk of rehospitalization. Considering the pivotal role of oxidative metabolism, hematologic homeostasis, and iron metabolism in HF pathophysiology and personalized risk stratification, we aimed to investigate the role of various genes involved in these processes.

The Haptoglobin gene (*HP*) encodes the haptoglobin protein (Hp). Through its antioxidant function, hemoglobin (Hb) binding properties, ability to stimulate macrophages and monocytes and to modulate T-cells, Hp prevents the loss of iron and Hb-mediated organ damage [8]. The complex Hp-Hb binds to receptor CD163 of monocytes and macrophages, and, after endocytosis, iron is liberated by the heme oxygenase enzyme [9]. Differences in the functionality of Hp have been observed between the protein products of the alleles of this gene (Hp1 and Hp2) in guarding against Hb-driven oxidative stress. Individuals with genotype Hp(1-1) present maximum efficiency and concentration while individuals with genotype Hp(2-2) have the minimum efficiency and concentration [10].

The angiotensin I converting enzyme gene (*ACE*)* *encodes the angiotensin-converting enzyme (ACE). The ACE is a key component of the Renin-Angiotensin-Aldosterone System, catalyzing the conversion of angiotensin I to angiotensin II. In this study, we focused on the I/D polymorphism. The analysis of this polymorphism (the presence of the larger insertion (I) allele or the shorter deletion (D) allele) showed that the alleles are codominant and impact the levels of plasmatic ACE with homozygotes for the I allele having the lowest levels of the enzyme, heterozygous individuals having an intermediate level and homozygotes for the D allele having the highest levels [11].

The methylenetetrahydrofolate reductase gene (*MTHFR*) encodes the methylenetetrahydrofolate reductase enzyme (MTHFR). This enzyme catalyzes the conversion of 5,10-methylenetetrahydrofolate to 5-methyltetrahydrofolate, a co-substrate for the remethylation of homocysteine to methionine. The polymorphism studied in this project was the C677T which consists of a C>T nucleotide modification at position 677. Individuals with genotype TT have around 30% of MTHFR enzyme activity and heterozygous individuals (CT) have around 65% enzyme activity when compared to people without the mutation [12]. The reduced activity of the MTHFR enzyme due to this mutation has been associated with different diseases such as hyperhomocysteinemia, coronary artery disease (CAD), thrombosis, or arterial hypertension. Elevated homocysteine levels will lead to the generation of ROS and induce oxidative stress in vascular cells, with homocysteine auto-oxidation contributing to the production of superoxide and hydrogen peroxide [13].

The homeostatic iron regulator gene (*HFE*) encodes the human homeostatic iron regulator protein, which regulates the interaction of transferrin receptors 1 and 2 with transferrin, regulating iron absorption. The C282Y is a missense variation of the *HFE* gene, caused by the mutation cysteine>tyrosine at amino acid 282. It has been found that C282Y homozygotes display higher levels of serum iron, transferrin saturation, serum ferritin levels [14], reduced hepcidin responsiveness to iron, and hepcidin deficiency [15]. The H63D is a missense variation caused by the substitution of histidine to aspartic acid at amino acid position 63. Like the C282Y mutation, it has also been related to higher levels of iron. Homozygous individuals for the H63D polymorphism generally exhibit higher iron indexes when compared to the wild type [16].

The cytochrome b-245 alpha chain gene (*CYBA*) encodes the p22^phox^ protein. The p22^phox^ protein is a subunit of the nicotinamide adenine dinucleotide (NAD) + hydrogen (H)/nicotinamide adenine dinucleotide phosphate (NADH/NAD(P)H) oxidase system, which is the main source of superoxide anion (O_^2^_^-^) in human blood vessels. The polymorphism we studied, C242T, consists of the substitution of histidine for tyrosine at position 242. It has been suggested that allele T causes a decreased NAD(P)H oxidase reactivity [17]. In line with this proposition, this variation would lead to a decreased production of ROS and oxidative stress in vessels [18]. The effect of this mutation on the development of cardiac disease and atherosclerosis remains unclear with controversial results [19-21].

Our study aims to evaluate the contribution of genes *HP*, *ACE*, *MTHFR*, *HFE*, and *CYBA* to HF development, independently or in epistasis, and thus identify specific interactions that may provide protection or susceptibility to the disease, to better characterize the genetic background of HF.

## Materials and methods

Study participants

We used samples from 389 individuals for our analysis. Genotype data were obtained from 143 HF patients, with a median age of 69.0 years (ranging from 31 to 94), and 246 healthy individuals, with a median age of 58.2 years (ranging from 18 to 80). The HF patients were followed in an internal medicine department of a tertiary care university hospital. 

Inclusion Criteria

Patients with HF admitted to an internal medicine ward of a tertiary care university hospital.

Exclusion Criteria

Patients with a glomerular filtration rate (GFR) less than 30 mL/min/1.73 m^2^ or undergoing renal replacement, with moderate to severe liver failure (according to the Child-Pugh score), in-hospital death during the first hospitalization, patients with hospital discharge against medical advice, and patients with cancer were excluded from the study.

The control group was provided by the Faculty of Human Motricity of the University of Lisbon consisting of individuals included in an exercise program from this Faculty. Individuals with arterial hypertension, hyperthyroidism, structural and/or functional heart diseases, or osteoporosis were excluded from the control group.

DNA extraction

Whole blood samples were obtained from patients and controls. Genomic DNA was isolated from total blood samples, using the NZY Tissue gDNA isolation kit, following the manufacturer's instructions.

*HP* genotyping

*HP* genotypes were determined by Hp phenotype (Hp-Hb complexes) in polyacrylamide gel electrophoresis (PAGE) and peroxidase staining using a modified version of Linke (1984) [22].

*ACE* genotyping

Polymerase chain reaction (PCR) amplification, flanking the I/D (Insertion-Deletion) variation, was performed using the primers 5’-GCCCTGCAGGTGTCTGCAGCATGT-3’ and 5’- GGATGGCTCTCCCCGCCTTCTCTC-3’. PCR conditions were: 30 cycles of 60 sec of 94ºC; 60 sec of 60ºC; 60 sec of 72ºC. Amplicons were visualized in agarose gel where 319 bp represented the allele with the deletion (D) and 597 bp represented the allele with the insertion (I).

*MTHFR* genotyping

PCR amplification, flanking the C677T variation, was performed using the primers 5’-TGAAGGAGAAGGTGTCTGCGGGA-3’ and 5’-AGGACGGTGCGGTGAGAGTG-3’. PCR conditions were: 35 cycles of 30 sec of 94ºC; 30 sec of 61ºC; 45 sec of 72ºC. An amplicon of 198 bp was visualized in agarose gel. Then, restriction with *Hinf *I allowed the following genotyping: CC - 198 bp; CT - 198, 175, and 23 bp; TT - 175 and 23 bp.

*HFE* genotyping

Sense primers for C282Y (5’-TGGCAAGGGTAAACAGATCC-3’) and H63D (5’-ACATGGTTAAGGCCTGTTGC-3’) were used in combination with the following specific antisense ARMS primers: C282Y normal/mutant 5'-GCTGATCCAGGCCTGGGTGCTCCACCTGCC/T-3' and H63D normal/mutant 5'-AGTTCGGGGCTCCACACGGCGACTCTCAAG/C-3' in two reaction tubes. Amplification was initiated by hot start after 4 min at 94ºC, followed by 30 cycles of 1 min at 94ºC, 59ºC, and 72ºC. Amplicons were visualized in agarose gel where two bands of 309 bp and 171 bp were used to genotype according to Baty et al. [23].

*CYBA* genotyping

PCR amplification, flanking the C242T variation, was performed using the primers 5’-TGCTTGTGGGTAAACCAAGGCCGGTG-3’ and 5’-AACACTGAGGTAAGTGGGGGTGGCTCCGT-3’. PCR conditions were: 35 cycles of 43 sec of 94ºC; 1 min of 54ºC; and 30 sec of 72ºC. An amplicon of 348 bp was visualized in an agarose gel. Then, restriction with *Rsa*I allowed the following genotyping: CC - 348 bp; CT - 348, 188, and 160 bp; TT - 188 and 160 bp.

Data analysis

Data analysis was conducted using SPSS® 26.0 software (IBM Corp., Armonk, NY, USA). In order to assess normality, the Kolmogorov-Smirnov test was used (n>30). In the presence of at least one group that did not follow a normal distribution, the Mann-Whitney U non-parametric test was used to compare the distribution regarding age. The χ2 test was used to compare the distribution regarding gender. Genotypic distributions between patient and control groups and epistatic interactions were analyzed using the χ2 and Fisher tests. Odds ratios (OR) were calculated as well as the corresponding confidence intervals (CI) with a 95% level of confidence. Results were then adjusted for age and gender using binary logistic regression. All results were considered to be statistically significant for a p-value < 0.05. Not all samples were made available for all genotyping protocols and, since samples are now categorized as archived samples, it was not possible to re-collect biological material from the same individuals. This explains why the number of samples (N) differs in each gene analysis. Likewise, for the sociodemographic characterization, only available data was used, leading to variations in the total sample size (N) for each variable (such as the New York Heart Association (NYHA) classification and type of HF).

Ethical considerations

Patients were identified with numeric codes in the database, and no personal information was collected. The costs of the tests were fully covered by the authors. Ethical principles of the Declaration of Helsinki were followed. All patients gave their informed consent to this study. The usage of patient data was approved by the Ethics Committee of the Academic Medical Center.

## Results

Sociodemographic variables

We assessed 143 patients with HF, the majority of whom had acutely decompensated HF. Age ranged from 31 to 94 years with a mean age of 69.0 years. Male gender accounted for 54.5% and female gender for 45.5% of individuals in the patient group. Regarding symptom classification, the majority of patients were classified as NYHA III (54.5%) or NYHA IV (35.0%).

Concerning ejection fraction, the majority of patients had reduced ejection fraction (82.9%) (Table 1). The individuals in our patient group presented the following comorbidities and/or risk factors: arterial hypertension, atrial fibrillation, diabetes mellitus type 2, ischemic cardiomyopathy, hypertensive cardiomyopathy, dilated cardiomyopathy, and systemic lupus erythematosus.

**Table 1:** Sociodemographic and clinical variables of the patient group NYHA: New York Heart Association; HF: Heart Failure; HFrEF: Heart Failure with Reduced Ejection Fraction; HFmrEF: Heart Failure with Mildly Reduced Ejection Fraction; HFpEF: Heart Failure with Preserved Ejection Fraction.

Variables		Mean	Standard Deviation
Age, mean (years)		69.0	11.9
Ejection Fraction (%)		35.4	10.0
		N	%
Sex	Male	78	54.5
	Female	65	45.5
Race	White	128	89.5
	Black	4	2.8
	Other	11	7.7
NYHA Classification	I	4	3.3
	II	9	7.3
	III	67	54.4
	IV	43	35.0
Type of HF	HFrEF	97	82.9
	HFmrEF	9	7.7
	HFpEF	11	9.4

The control group consisted of 246 healthy individuals, 45 males (18.3%) and 201 females (81.7%). The mean age of individuals in the control group was 56.5 years. None of these individuals presented with any of the listed comorbidities or risk factors.

One gene analysis

The only gene associated with the disease was *MTHFR*. We observed risk for genotype CT, using CC as reference (OR (CI, 95%) = 3.265 (1.351-7.894); p=0.009 (adjusted to age and sex)). Additionally, we observed that the presence of allele T (Dominant Model) constituted a risk for the development of HF (OR (CI, 95%) = 2.932 (1.210-7.101); p=0.017 (adjusted to age and sex)). Furthermore, our analysis shows that having only one T allele (CT) (overdominant model) was sufficient to be at risk (OR (CI, 95%) = 3.618 (1.507-8.687); p=0.004 (adjusted for age and sex)) (Table 2).

**Table 2:** Analysis of the C677T polymorphism of the MTHFR gene OR: odds ratio; CI,95%: 95% confidence interval; ^1^ χ2 Pearson; ^2^ Fisher’s Exact Test; ^3^ Adjusted for age and sex using binary logistic regression.

Genetic Models	Genotypes	Controls N (%)	Patients N (%)	p-value		OR (CI, 95%)	
				Unadjusted	Adjusted (age, sex)	Unadjusted	Adjusted (age, sex)
Codominant	CC	68 (48.6)	15 (23.4)	<0.001^1^	Reference		
	CT	62 (44.3)	49 (76.6)		0.009^3^	3.583 (1.828-7.023)	3.265^3^ (1.351-7.894)
	TT	10 (7.1)	0 (0.0)		0.999^3^	-	-
Recessive	TT	10 (7.1)	0 (0.0)	0.033^2^	0.999^3^	-	-
	CC+CT	130 (92.9)	64 (100.0)				
Overdominant	CT	62 (44.3)	49 (76.6)	<0.001^1 ^	0.004^3^	4.110 (2.108-8.012)	3.618^3^ (1.507-8.687)
	CC+TT	78 (55.7)	15 (23.4)				
Dominant	TT+CT	72 (51.4)	49 (76.6)	<0.001^1^	0.017^3^	3.085 (1.584-6.009)	2.932^3^ (1.210-7.101)
	CC	68 (48.6)	15 (23.4)				

Epistatic analysis

The risk was observed for the interaction between genotype II of the *ACE *gene and genotype CC of the polymorphism C282Y (*HFE*) (OR (CI, 95%) = 5.191 (1.582-17.031); p=0.007 (adjusted for age and sex)) and between genotype II of the *ACE* gene and genotype HH of polymorphism H63D (*HFE*) (OR (CI, 95%) = 6.626 (1.658-26.473); p=0.007 (adjusted for age and sex)) (Table 3).

**Table 3:** Analysis of the epistatic interactions between genes ACE and HFE OR: odds ratio; CI,95%: 95% confidence interval; *ACE*: angiotensin I converting enzyme gene; *HFE*: homeostatic iron regulator gene; ^1^ χ2 Pearson; ^2^ Adjusted for age and sex using binary logistic regression.

Polymorphisms In epistasis	Genotype interaction	Controls N (%)	Patients N (%)	p-value		OR (CI, 95%)	
				Unadjusted	Adjusted (age, sex)	Unadjusted	Adjusted (age, sex)
I/D(*ACE*)–C282Y(*HFE*)	II - CC	13 (10.2)	11 (15.9)	0.244^1^	0.007^2^	1.663 (0.702-3.942)	5.191^2^ (1.582-17.031)
	Other combinations	114 (89.8)	58 (84.1)				
I/D(*ACE*) – H63D(*HFE*)	II - HH	10 (7.9)	10 (14.5)	0.149^1^	0.007^2^	1.966 (0.775-4.987)	6.626^2^ (1.658-26.473)
	Other combinations	116 (92.1)	59 (85.5)				

We also obtained risk for the interactions between genotype CC of the *CYBA* gene and genotypes 2-2 (*HP*) (OR (CI, 95%) = 3.467 (1.185-10.142); p=0.023 (adjusted for age and sex)), CT (*MTHFR*) (OR (CI, 95%) = 5.480 (1.926-15.590); p=0.001 (adjusted for age and sex)) and HH (*HFE-H63D*) (OR (CI, 95%) = 3.240 (1.222-8.588); p=0.018 (adjusted for age and sex)) (Table 4).

**Table 4:** Analysis of the epistatic interactions between CYBA gene and HP, MTHFR and HFE genes OR: odds ratio; CI,95%: 95% confidence interval; *CYBA*: cytochrome b-245 alpha chain gene; *HP*: haptoglobin gene; *MTHFR*: methylenetetrahydrofolate reductase gene; *HFE: *homeostatic iron regulator gene ^1^ χ2 Pearson; ^2^ Adjusted for age and sex using binary logistic regression.

Polymorphisms In epistasis	Genotype interaction	Controls N (%)	Patients N (%)	p-value		OR (CI, 95%)	
				Unadjusted	Adjusted (age, sex)	Unadjusted	Adjusted (age, sex)
C242T(*CYBA*) –1,2(*HP*)	CC – (2-2)	10 (11.6)	15 (21.1)	0.105^1^	0.023^2^	2.036 (0.852-4.866)	3.467^2^ (1.185-10.142)
	Other combinations	76 (88.4)	56 (78.9)				
C242T (*CYBA*) – C677T (*MTHFR*)	CC – CT	21 (19.3)	20 (47.6)	<0.001^1^	0.001^2^	3.810 (1.763-8.230)	5.480^2^ (1.926-15.590)
	Other combinations	88 (80.7)	22 (52.4)				
C242T (*CYBA*) – H63D (*HFE*)	CC – HH	14 (17.7)	26 (40.0)	0.003^1^	0.018^2^	3.095 (1.446-6.628)	3.240^2^ (1.222-8.588)
	Other combinations	65 (82.3)	39 (60.0)				

## Discussion

The individual analysis of the *MTHFR *gene* *emphasizes that individuals with allele T display a higher risk of developing HF than those without this allele. These results corroborate what we expected since individuals with allele T exhibit reduced enzyme activity and higher levels of homocysteine, contributing to elevated oxidative stress and increased susceptibility to atherosclerosis and vascular disease.

In our study, the epistatic interactions between genotype II (*ACE*) and genotypes CC and HH (both in *HFE*) represented a risk for HF. Individuals with the wild-type genotype CC (*HFE-C282Y*) generally have lower iron levels than those with the YY genotype, which predisposes them to hereditary hemochromatosis [24]. The wild-type genotype of polymorphism H63D (genotype HH) is also associated with lower levels of iron-related biomarkers than other genotypes [25]. One previous study suggested that the C282Y polymorphism may have represented an adaptive advantage compensating for dietary iron deficiency [26]. Another study proposed that women inheriting heterozygosity for the C282Y polymorphism may be less likely to present iron deficiency compared to women with the CC genotype [27]. Consequently, when combined with other factors such as a low-iron diet, the CC and HH genotypes in *HFE *may predispose individuals to iron deficiency and anemia. On the other hand, angiotensin II increases the production of erythropoietin. With genotype II leading to lower levels of *ACE*, the production of angiotensin II and, consequently, erythropoietin, will be lower. Considering that these genotypes result in reduced erythropoiesis and lower iron levels, we propose that their combination favors iron deficiency and ferropenic anemia, an aggravating factor of HF, and represents a risk for developing the disease.

Controversy exists as to whether the C242T polymorphism of the *CYBA *gene affects the susceptibility to HF. Seeking to clarify the role of this polymorphism in heart disease, we noted a consistent correlation between genotype CC and risk for HF, when associated with other genes (*HP*, *MTHFR, *and *HFE*) in epistasis. This suggests that, even though its isolated presence may not be a clear risk factor for HF, it may represent susceptibility to disease when simultaneously present with certain other genotypes.

Superoxide production appears to be higher in patients with allele C of C242T than baseline superoxide production among the individuals who do not carry allele C [18]. The superoxide anion - O^2-^ - plays a crucial role in oxidizing LDL and exacerbating inflammation in atherosclerotic plaques. Pro-oxidant agents and oxidative stress coincide with increased lipid levels and endothelial dysfunction. The risk observed for the interaction between genotype CC and genotype 2-2 is justified by the dual role in oxidative stress presented by these two genotypes. Genotype 2-2 is associated with less protection against hemoglobin iron-driven peroxidation as well as increased levels of oxidized LDL [28], representing a risk for atherosclerosis and the development of CAD. When associated with the CC genotype of the *CYBA *gene, the cumulative result of these pro-oxidative effects presented by both variants results in risk for developing HF.

We also observed risk between genotype CC (*CYBA*) and genotype CT of the *MTHFR *gene which, alone, was seen to present a risk for HF (OR (CI, 95%) = 3.618 (1.507-8.687); p= 0.004 (adjusted for age and sex)). When in epistasis, this risk increased to 5.480 (OR (CI, 95%) = 5.480 (1.926-15.590); p=0.001 (adjusted for age and sex)). With no patients presenting genotype TT, the contribution of allele T is mostly assessed through the analysis of genotype CT. Individuals with allele T normally display higher levels of homocysteine. As stated before, when homocysteine levels are higher, there is an increase in ROS production in vascular cells, thereby directly relating high homocysteine levels to oxidative stress in myocardial ischemia/reperfusion injury [13]. Combined with a higher superoxide production also verified in the presence of genotype CC of the *CYBA* gene, this interaction exacerbates oxidative damage and further favors atherosclerosis, justifying the observed rise in risk.

We found out that the interaction between genotype CC (in *CYBA*) and genotype HH of the H63D polymorphism (*HFE*) represented a risk. Homozygous individuals for allele H generally exhibit lower levels of iron and ferritin, when compared to the other genotypes [25]. Ferritin behaves as an endogenous antioxidant by sequestering toxic labile iron. Iron deficiency leads to impaired metabolism of ROS [2], affecting the redox state, and can be reversed by supplementation and the inclusion of antioxidants in treatment strategies, which seems to be successful in lessening the damage caused by oxidative stress. Iron deficiency and anemia promote a state of hypoxia which aggravates the oxidative activity already altered by the increased production of superoxide through genotype CC in *CYBA *and by shifts in cellular metabolism and activation of leukocytes which ultimately lead to more production of free radicals and oxidation [29].

Limitations

For future studies, we believe that a better characterization of the patient population, especially regarding therapeutics and diet, would be advantageous. Bearing in mind that genetic polymorphisms vary among different ethnicities and populations, the interpretation of our findings must take into consideration the sociodemographic variables of our population. Another limitation that we identified was the absence of the determination of the serum biomarkers of iron homeostasis so that they could be assessed in conjunction with the genotypes of the *HFE *gene. We consider that the size of our sample also represented a limitation; in fact, larger populations would certainly provide us with more reliable results. In addition, a multi-center study will improve the ability to generalize our results. Nevertheless, we used data from a real-world clinical practice setting and obtained results that are in line with and complementary to previously published studies, thus supporting the contribution of our findings.

## Conclusions

Our investigation reveals that genes *HP*, *ACE*, *MTHFR*, *HFE, *and *CYBA *contribute to the susceptibility of developing HF, either individually or in epistasis. Through our one-gene and epistatic analyses, we concluded that genotypes which, in a particular context, induce higher oxidative stress (2-2 (*HP*), CT (*MTHFR*) and CC (*CYBA*)) and affect the indexes of iron (CC and HH (*HFE*)) or hematologic homeostasis (II (*ACE*)) are associated with an increased risk of developing HF. This study is in line with the ESC Heart Failure Guidelines recommendations that the study of iron and hematologic status should be performed in patients with HF. By shedding light on and contributing to the clarification of the roles of genes involved in the metabolism of iron and oxidative mechanisms in the pathophysiology of HF, we believe that this investigation paves the way for the study of antioxidant therapies in the treatment of HF and represents a promising step forward in risk stratification and tailored medicine.
